# Serum Ferritin in Obese Dogs: Changes and Comparison with Other Analytes

**DOI:** 10.3390/vetsci10070457

**Published:** 2023-07-11

**Authors:** Lorena Franco-Martínez, Luis Pardo-Marín, Laura Sánchez-Mateos, Alberto Muñoz-Prieto, Juan Diego García-Martínez, José J. Cerón, Silvia Martínez-Subiela, Camila P. Rubio, Asta Tvarijonaviciute

**Affiliations:** 1 Interdisciplinary Laboratory of Clinical Pathology, Interlab-UMU, Campus of Excellence Mare Nostrum, University of Murcia, 30100 Murcia, Spain; lorena.franco2@um.es (L.F.-M.); lpm1@um.es (L.P.-M.); alberto.munoz@um.es (A.M.-P.); juandi@um.es (J.D.G.-M.); jjceron@um.es (J.J.C.); silviams@um.es (S.M.-S.); asta@um.es (A.T.); 2 Moorepark Animal and Grassland Research Center, Teagasc, Irish Agriculture and Food Development Authority, P61 C996 Cork, Ireland; 3 Clínica Veterinaria Dinos, Espinardo, 30100 Murcia, Spain; dinosclinicavet@gmail.com

**Keywords:** acute phase proteins, biomarkers, canine obesity, dog, ferritin, hypoxia, inflammation

## Abstract

**Simple Summary:**

Canine obesity, defined as a weight greater than 15% of the ideal, is associated with decreased quality of life, longevity, and other complications. However, the mechanisms causing these alterations are not fully understood. In obese humans, increased concentrations of ferritin in serum have been found; however, this has been poorly explored in dogs. In this study, ferritin levels from lean/normal weight (CG, *n* = 55) and overweight/obese dogs (OG, *n* = 37) were evaluated, together with complete hemogram and biochemical analyses. Higher ferritin concentrations were observed in the OG group in comparison to the CG. The increases in ferritin, together with higher haematocrit and erythrocyte mean corpuscular volume, could indicate tissue hypoxia in obese dogs.

**Abstract:**

Canine obesity is the most common nutritional disorder and is associated with decreased quality of life and longevity as well as comorbidities including cardiorespiratory, endocrine, oncologic, or orthopaedic disorders. Ferritin is a major acute-phase protein in dogs, increasing during inflammation; however, it could also be affected by other conditions, including trauma, iron metabolism dysregulations, neoplasia, or hypoxia. Higher ferritin levels have been reported in obese humans, but ferritin has not been explored in canine obesity. To evaluate the possible changes in serum ferritin in canine obesity, ferritin levels from lean/normal weight (CG, *n* = 55) and overweight/obese dogs (OG, *n* = 37) were measured, together with complete hemogram and biochemical analyses. Statistically significant higher ferritin levels (1.2-fold) were found in OG (median, (interquartile range), 204 (166–227.5) µg/L) in comparison to CG animals (172 (137–210) µg/L)), with median levels of ferritin in OG dogs above the reference range for healthy animals in our laboratory (60–190 µg/L). In addition, statistically significant higher mean corpuscular volume (MCV), mean cell haemoglobin concentration (MCHC), total proteins, globulins, haptoglobin, total ferric fixation capacity (TIBC), alkaline phosphatase (ALP), butyrylcholinesterase (BChE), triglycerides, and calcium were observed in OG in comparison to CG. The higher levels in ferritin, together with higher TBIC, haematocrit, and MCV, could indicate tissue hypoxia in obese dogs.

## 1. Introduction

Canine obesity is the most common nutritional disorder [1] characterized by the accumulation of excess adipose tissue in the body and defined in dogs as weight greater than 15% of the ideal. Overweight and obese dogs can reach a prevalence higher than 50% of the population in some places [2,3,4]. Canine obesity is associated with decreased quality of life and longevity and is related to the development of comorbidities such as cardiorespiratory, endocrine, oncologic, or orthopaedic disorders [5,6]. However, although dogs with obesity present insulin resistance, increased blood lipids, altered levels of adipokines, and oxidative stress markers in comparison to control ones, the pathophysiology of canine obesity is still unclear [7].

Ferritin is a protein that stores iron within cells and facilitates its transport between tissues. Circulating levels of ferritin reflect that iron storage levels are being affected by iron metabolism dysregulations [8,9]. Furthermore, ferritin circulating concentrations are increased during inflammatory response [10], due to traumatism which leads to cellular damage and rupture [11], neoplasia [12], or hypoxia [13], among others. In humans, increased ferritin levels cluster with obesity [14] and with a higher risk of metabolic syndrome [15,16,17,18] and its components, such as dyslipidaemia [19], high blood pressure [20], and type 2 diabetes [15,21]. However, to the best of the authors’ knowledge, the relationship between serum ferritin levels and canine obesity is still not elucidated, except for a single article [22] that reported higher, although not statistically relevant, serum ferritin in obese dogs with obesity-related metabolic dysfunction (ORMD) in comparison to non-ORMD obese dogs.

The aim of this study was to evaluate the possible changes in serum ferritin in canine obesity. In addition, a complete blood count and serum biochemistry, including a panel of acute-phase proteins (APPs) with a major (CRP), a moderate (haptoglobin), and negative (albumin, PON-1) APPs, were measured for comparative purposes and to gain information that elucidates possible changes in ferritin levels. Besides the common haematological and biochemical panel, other analytes that could provide additional information were also evaluated. Therefore, iron metabolism was assessed by the measurement of iron, ferritin, total iron-binding capacity (TIBC), unsaturated iron-binding capacity (UIBC), and ferritin saturation. TIBC measures the maximum amount of iron that can be bound to transferrin. UIBC indicates the amount of transferrin available to bind more iron, and ferritin saturation represents the percentage of transferrin that is currently bound to iron. In addition, paraoxonase-1 (PON-1) and butyrylcholinesterase (BChE) were measured. PON-1 is an enzyme with esterase activity that is physically bound to high-density lipoprotein (HDL), and that decreases in inflammatory processes, intestinal tract disorders, or human obesity [23,24,25]. BChE increases significantly in overweight/obese dogs [26].

## 2. Materials and Methods

### 2.1. Animals and Samples

The study was a retrospective study approved by the University of Murcia and the Murcia Region Ethical Committee (Number, A13170503; Date, 27 September 2022).

Serum surplus from lean, normal weight, overweight, and obese client-owned dogs presented at veterinary clinics of the Murcia Region for routine check-ups or vaccinations and submitted to the Clinical Pathology Laboratory Interlab-UMU for CBC and serum biochemistry between January 2020 and January 2022 were assessed for inclusion in the study.

Inclusion criteria for the overweight/obese group (OG) were being an overweight or obese but otherwise healthy adult dog (body condition score (BCS) between 6 and 9 on a 9-point body condition scale chart (BCS 6–9/9) [27] with a history of more than one year, according to clinical examination, CBC, and biochemical analysis. None of the dogs of the OG presented ORMD, according to previous literature [28]. The lean/normal-weight group (control group, CG) was selected to match the OG group in terms of age, breed, and size. In addition, they had to fulfil the following inclusion criteria: to be a slightly lean or normal weight (BCS 4–5/9) adult and present no abnormalities upon clinical examination, CBC, and biochemical analysis.

All animals had a negative serological titter for *Leishmania infantum* and *Ehrlichia canis*, as demonstrated by commercial tests (Canine SNAP tests, IDEXX Europe B.V., Hoofddorp, The Netherlands).

### 2.2. Analysis

Ferritin was measured using commercially available reagents (Olympus, Beckman Coulter, Fullerton, CA, USA) following the manufacturers’ instructions using an automated analyser (Olympus AU600, Beckman Coulter).

Complete blood count analyses were performed. The red fraction was analysed for red blood cells (RBC); haematocrit; haemoglobin; mean corpuscular volume (MCV); mean corpuscular haemoglobin (MCH); mean cell haemoglobin concentration calculated from the optically measured cellular haemoglobin (MCHC); and red cell distribution width (RDW). The white blood fraction was analysed for white blood cell count (WBC) and segmented lymphocyte, monocyte, eosinophil, and basophil counts. Platelets were assessed for platelet count (platelets); mean platelet volume (MPV); platelet crit (PCT); mean platelet component concentration (MPC); and platelet component concentration distribution width (PCDW). All analyses were performed using an automated analyser (ADVIA 120 haematology Analyzer; Siemens Healthcare GmbH; Erlangen; Germany).

The following parameters were measured for serum biochemistry using commercially available reagents (Olympus, Beckman Coulter) and following the manufacturer’s instructions: total proteins, albumin, C-reactive protein (CRP), creatinine kinase (CK), aspartate aminotransferase (AST), alanine aminotransferase (ALT), alkaline phosphatase (ALP), gamma glutamyl aminotransferase (gGt), total bilirubin, total cholesterol, triglycerides, amylase, urea, creatinine, glucose, calcium, and phosphorus. Globulin concentration was calculated by subtracting the concentration of albumin from the total protein concentration. Serum butyrylcholinesterase (BChE) activity was measured using butyryl thiocholine iodide as a substrate [26]. Serum paraoxonase-1 (PON-1) activity was determined using p-nitrophenyl acetate [29]. Total antioxidant capacity (TAC) and total oxidative status (TOS) were measured using colorimetric assays [30,31,32]. Haptoglobin concentration was measured using a commercially available method (Tridelta Phase Range Haptoglobin Kit; Tridelta Development [33]. All analyses were performed in an automated analyser (Olympus AU600, Beckman Coulter).

### 2.3. Statistical Analysis

The normality of the data’s distribution was assessed for each biomarker using the D’Agostino and Pearson omnibus normality test. Then, since most analytes presented a non-Gaussian distribution, Mann–Whitney U tests were performed to evaluate possible differences between the two groups. A non-parametric Spearman correlation test was used to assess possible correlations between ferritin and the other parameters. Values of *P* < 0.05 were considered as statistically significant. Analyses were performed using the statistical software GraphPad Prism 8.

## 3. Results

The study included a total of 92 dogs. The baseline characteristics of the animals involved in the study are presented in Table 1. There were no statistically significant differences between the groups in terms of sex, age, tartar, gingival inflammation, feed type, meals per day, or whether they were receiving supplements or snacks. However, the OG had a higher percentage of sterilised dogs (73% versus 40%), body weight, and BCS than CG (*P* < 0.05 in all cases). Data in bold highlight statistical significance.

**Figure 1:**
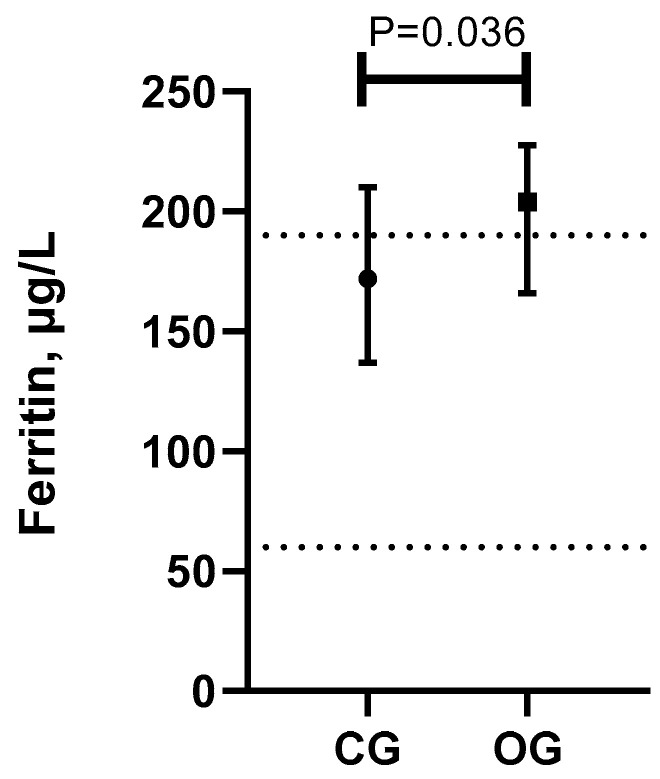
Serum ferritin (median, 25–75th percentile) levels in lean/normal weight (control group, CG) and overweight/obese (obese group, OG) dogs. Grid lines represent the reference range for healthy animals in our laboratory (60–190 µg/L).

Ferritin was 1.2-fold higher in obese dogs (median, (25–75th interquartile range), 204 (166–227.5) µg/L) in comparison to lean/normal weight animals (172 (137–210) µg/L)) (*P* < 0.05), with median levels of ferritin in OG dogs above the reference range for healthy animals in our laboratory (60–190 µg/L) (Figure 1).

The haematological analyses revealed that dogs from OG had 1.02-fold higher MCV values but 1.03-fold lower MCHC values than lean/normal-weight dogs (Table 2). The biochemical analysis (Table 3) also showed differences between the two groups. OG dogs had 1.2-fold higher levels of haptoglobin, TIBC, and BChE; 1.6-fold higher triglycerides; 1.4-fold higher ALP; and 1.1-fold higher total proteins, albumin, globulins, and calcium in comparison to CG. Haptoglobin, TIBC, and BChE medians were above the reference range of the laboratory in OG.

Correlations between ferritin and the rest of the analytes were calculated for all dogs and for each group separately (CG and OG) and are represented in Table 4. When data from all animals were pooled, a statistically significant correlation was observed between ferritin and AST and gGT. In CG, a positive statistically significant correlation was found between ferritin and MCHC and a negative one between ferritin and gGT. In the OG group, ferritin was positively significantly correlated with MCV and MCH and negatively with RDW, WBC, and eosinophils. 

## 4. Discussion

In this study, the effect of obesity on serum concentrations of ferritin was investigated. In addition, a CBC and serum biochemistry that included a panel of acute phase proteins were performed with the aim of providing new insights into canine obesity pathology.

Serum ferritin levels were statistically higher in OG in this report; this agrees with human studies that described an increase in ferritin in obese individuals [18,19] and suggested that this increase is due to different pathways that could be related to inflammation and hypoxia [34,35].

Regarding the possible causes for increased ferritin in OG in our study, there are some that could be ruled out based on the CBC and biochemistry results. Serum iron levels, haemoglobin, UIBC, and ferric saturation did not show increases in the obese dogs of our report; therefore, iron metabolism dysregulation could be ruled out as a possible reason for altered ferritin levels in OG. In a similar manner, it is unlikely that those overweight/obese animals presented muscular damage since the levels of AST and CK were within the reference interval and did not differ significantly from CG. Hepatic enzymes (ALT, AST, and ALP), bilirubin, and hepatic proteins (globulins and albumin) did not show alterations of interest, thus exhibiting adequate hepatic function. None of the dogs were reported to have had recent trauma, and no evidence of neoplasia or immunological disorders was observed in the physical examination, CBC, or biochemistry analysis.

In addition, the presence of active inflammation as a cause of increased ferritin could be discarded based on our results on APPs. Ferritin is a positive moderate APP in dogs [36]. However, there is controversy regarding the presence of inflammation associated with canine obesity. Obesity can cause inflammation through different pathways [34,35,37,38]. The excessive adipose tissue increases the secretion of pro-inflammatory cytokines and APPs, thus promoting a chronic and mild inflammatory state. Additionally, changes in the intestinal microbiome promote the exacerbation of certain bacterial species that produce proinflammatory metabolites. In dogs, there are limited and contradictory data regarding alterations in APPs in canine obesity. One study links obesity with increased levels of CRP, plasma tumour necrosis factor-alpha, and haptoglobin, with a reduction in these parameters after weight loss [39], while others report decreased CRP [40] or no difference in APPs [28,41,42]. In our study, CRP and PON-1 were unaltered, while haptoglobin and albumin were higher in OG. Interestingly, this profile was described in dogs diagnosed with a situation of hypercortisolism such as hyperadrenocorticism [43,44,45]. These studies reported increases in haptoglobin but no significant changes in CRP or albumin in dogs with uncomplicated hyperadrenocorticism in comparison to healthy dogs [43]; in addition, haptoglobin was reduced after hyperadrenocorticism control [45]. Furthermore, OG dogs in our study also presented higher ALP, triglycerides, albumin BChE, and lower lymphocytes (presenting a stress leukogram), thus further supporting the presence of hypercortisolism in dogs with obesity, as described in human obesity [46]. Nevertheless, the profile of APPs highlights that OG dogs did not show signs of active inflammation, which could explain the higher levels of serum ferritin observed in these dogs in comparison to CG ones. 

Another possible cause of the increase in serum ferritin could be the presence of tissular hypoxia. In these dogs, the TBIC, which is the maximum ferric concentration fixable by serum proteins, mainly transferrin, was also found to be increased. Previous studies had described elevated haemoglobin, ferritin and erythropoietin concentrations in humans with metabolic syndrome [38], and studies with obese and overweight dogs also support the possible existence of tissular hypoxia in obese dogs. A previous report pointed out a downregulation of haemoglobin subunits alpha- and beta-like genes in obese dogs [7]. Also, a decrease in arterial oxygen partial pressure and inspired oxygen fraction in obese sedated dogs, in comparison to the same animals after a weight loss program, has been described [47]. Therefore, based on our results and previous literature, we hypothesise that OG dogs may present tissue hypoxia, as described for humans, and this condition could have influenced the increase in ferritin serum levels; however, further studies would be needed to confirm this hypothesis.

This study presents some limitations. The use of client-owned dogs increases the variability in terms of breed, feed type and frequency, exercise, and age of onset of obesity, among other factors which may have influenced our results and should be further evaluated. In our study, the OG presented a higher rate of sterilization than the CG. However, this represents the true clinical picture. Second, in this study, data from obese dogs without ORMD were included. In the only study where serum ferritin levels were evaluated in canine obesity by comparing samples obtained from obese dogs with and without ORMD, the ORMD group dogs showed 1.2-fold higher circulating ferritin levels than obese non-ORMD, although this difference was not statistically relevant [22]. Although in our study, non-ORMD OG dogs presented slightly higher ferritin than that reported in the previous study (204 (166–227.5 µg/L) in our study versus 184.3 (145.4–247.1) µg/L in [22]), ferritin levels in non-ORMD are higher than in the normal weight and lower than in the ORMD dogs. Further analyses would be required to determine the possible causes of these differences in ferritin values in non-ORMD obese dogs between both studies, such as the possible presence of dogs that are closer to meeting ORMD diagnosis criteria. In any case, based on the results from both studies, ferritin is lower in lean/normal weight that in overweight/obese dogs without ORMD and obese dogs presenting ORMD, although the differences between the obese dogs with and without ORMD are of a lower magnitude. Finally, since punctual measurements of cortisol in the serum of dogs present high inter- and intra-individual variability, with one of the main reasons being the circadian fluctuations of this analyte [48,49]; it was not used in this study to confirm our hypothesis of hypercortisolism [46]. Other recommended alternatives, such as the ACTH stimulation test, the dexamethasone suppression test, or cortisol in 24 h urine, were not performed due to the retrospective nature of this work, but they could help to clarify the presence of a hypercortisolism state in overweight/obese dogs in future studies.

## 5. Conclusions

In the population of obese dogs of our study, there was an increase in serum ferritin associated with increases in haematocrit, TBIC, and MCV, which could be indicative of tissue hypoxia. In addition, a profile of APPs consisting of normal CRP, increased Hp, BChE, and ferritin is associated with canine obesity and could suggest the presence of a hypercortisolism state in this disease. Further studies should be performed to elucidate the possible application of ferritin alone or in combination with other APPs as biomarkers of obesity in dogs.

## Figures and Tables

**Table 1:** Descriptive data of the studied population.

Variable	Lean/Normal Weight (CG, *n* = 55)	Overweight/Obese (OG, *n* = 37)	*P*
Sex (female/male)	32/23	23/21	0.203
Sterilised, %	40	73	**0.002**
Age (Years) *	8 (6.6–11)	10 (7–10.5)	0.183
Body weight (Kg) *	5.525 (4.1–10.7)	8.4 (6.375–12.05)	**0.027**
BCS *	5 (4–5)	6 (6–7)	**0.000**
Tartar (%) *	55 (26.25–80)	65 (50–80)	0.449
Gingiva (0/1/2/3)	6/16/22/11	3/12/18/4	0.572
Meals per day (1/2/3/>3)	3/22/12/18	4/17/6/10	0.494
Supplements (Yes/No)	49/5	30/4	0.706
Snacks (Yes/No)	28/27	26/11	0.064

* Data are presented as median (interquartile range). BCS: body condition score.

**Table 2:** Haematology data.

Variable	Lean/Normal Weight (CG, *n* = 55)	Overweight/Obese (OG, *n* = 37)	*P* (CG vs. OG)	Reference
RBC (10^6^/µL)	7.28 (6.57–7.66)	7.23 (6.79–7.68)	0.605	5.69–8.56
Haemoglobin (g/dL)	17.3 (15.4–18.1)	17.4 (15.75–18.45)	0.251	13.7–20.6
Haematocrit (%)	49.4 (46–52)	51.9 (46.9–54.5)	0.052	37–58
MCV (fL)	69.6 (67.6–71)	71.4 (69.45–74.15)	**0.001**	61.7–74.1
MCH (pg)	23.7 (22.9–24.6)	24.2 (23.25–25.3)	0.263	21.4–25.5
MCHC (g/dL)	33.4 (32.4–34)	32.6 (32.1–33.1)	**0.001**	33.2–36.8
RDW (%)	11.5 (11.2–12)	11.8 (11.4–12.25)	0.091	11.3–15.1
WBC (10^3^/µL)	6.72 (5.77–8.12)	6.94 (5.89–8.615)	0.558	5.2–14
Segmented (10^3^/µL)	3.75 (3.148–4.79)	4.35 (3.66–5.36)	0.055	3.1–11
Lymphocytes (10^3^/µL)	2.09 (1.6–2.625)	1.8 (1.45–2.45)	0.119	1–3.8
Monocytes (10^3^/µL)	0.37 (0.28–0.525)	0.45 (0.275–0.525)	0.421	0.2–0.8
Eosinophiles (10^3^/µL)	0.325 (0.185–0.475)	0.28 (0.17–0.42)	0.305	0.1–1.4
Basophiles (10^3^/µL)	0.03 (0.01–0.04)	0.02 (0.0125–0.04)	0.897	0–0.03
Platelets (10^3^/µL)	347 (284.8–427)	345 (257–372)	0.382	175–588
MPV (fL)	10.2 (9.325–12.13)	10.8 (9.8–12.1)	0.214	8.6–15.8
PCT (%)	0.36 (0.29–0.4275)	0.35 (0.29–0.44)	0.732	0.2–0.8
MPC (g/dL)	21.55 (20.8–22.18)	21.65 (20.25–22.6)	0.590	21–100
PMDW (pg)	0.715 (0.6525–0.8)	0.8 (0.7–0.85)	0.088	0.64–1.07

Data are presented as median (25–75th interquartile range). RBC: red blood cells; MCV: mean corpuscular volume; MCH: mean corpuscular haemoglobin; MCHC: mean cell haemoglobin concentration; WBC: white blood cell count; MPV: mean platelet volume; PCT: platelet crit; MPC: mean platelet component concentration; PMDW: platelet dry mass distribution width. Data in bold highlight statistical significance. Internal laboratory reference values.

**Table 3:** Biochemistry data.

Variable	Lean/Normal Weight (CG, *n* = 55)	Overweight/Obese (OG, *n* = 37)	*P* (CG vs. OG)	Reference
Ferritin (µg/L)	172 (137–210)	204 (166–227.5) a	0.036	60–190
Total proteins (g/dL)	6.37 (6.06–6.76)	6.89 (6.655–7.37)	**<0.001**	5.4–7.2
Albumin (g/dL)	3.1 (2.9–3.4)	3.3 (3.1–3.65)	**0.007**	2.5–3.6
Globulins (g/dL)	3.3 (2.8–3.88)	3.5 (3.3–3.95)	**0.028**	2.6–3.8
CRP (µg/mL)	2.1 (1.6–4.8)	2.1 (1.45–4.3)	0.313	0–12
Haptoglobin (g/L)	3 (2.1–3.79)	3.6 (2.885–4.25) a	**0.031**	0–3
Fe (µg/dL)	134.8 (99.25–175.6)	153.6 (105.2–186.4)	0.456	81–198
UIBC (µg/dL)	254 (210.4–312.1)	274.7 (219.4–362)	0.386	148–311
TIBC (µg/dL)	373.7 (345.8–462.6)	452.8 (391.9–517) a	**0.019**	305–436
Ferritin saturation (%)	35.6 (23.93–43.84)	35.3 (25.01–46.37)	0.963	23.1–51.5
CK (IU/L)	104 (67–161)	99 (72.5–131)	0.962	30–360
AST (IU/L)	29 (22–34)	28 (21–35.5)	0.845	0–50
ALT (IU/L)	48 (36–70)	58 (38–89.5) a	0.205	0–50
ALP (IU/L)	79 (53–111)	112 (66.5–190)	**0.037**	25–190
gGT (IU/L)	4.8 (3.175–6.675)	3.9 (1.25–5.6)	0.133	1–6.5
Total bilirubin (mg/dL)	0.14 (0.1–0.18)	0.12 (0.11–0.16)	0.795	0.06–0.24
BchE (µmol/mL.min)	4.36 (3.5–5.6)	5.2 (4.25–8.05) a	**0.005**	3–5
PON-1 (IU/mL)	3.5 (3.1–3.9)	3.6 (3.15–4.15)	0.558	3–4.3
Cholesterol (mg/dL)	239 (207–282)	239 (212–340.5)	0.659	120–300
Triglycerides (mg/dL)	64 (55–89)	103 (69.5–185.5)	**<0.001**	30–200
Amylase (IU/L)	607 (448–795)	648 (517.5–791)	0.608	250–1300
Urea (mg/dL)	38.2 (31.3–47.9)	36.6 (30.9–46.3)	0.994	20–50
Creatinine (mg/dL)	0.9 (0.8–1)	0.9 (0.795–1.03)	0.864	0.5–1.5
Glucose (mg/dL)	101 (95–108)	96 (85.5–108.8)	0.052	70–110
Calcium (mg/dL)	9.91 (9.54–10.43)	10.76 (10.15–11.11)	**<0.001**	9.6–11.7
Phosphorus (mg/dL)	3.6 (3.22–4.2)	3.9 (3.65–4.45)	0.066	2.6–4
Ca/P	2.75 (2.3–3.225)	2.7 (2.4–2.9)	0.884	

PON-1: paraoxonase-1; CRP: C-reactive protein; Fe: iron; UIBC: unsaturated iron-binding capacity; TBIC: total ferric fixation capacity; CK: creatinine kinase; AST: aspartate aminotransferase; ALT: alanine aminotransferase; ALP: alkaline phosphatase; gGT: gamma glutamyl aminotransferase; BchE: butyrylcholinesterase; Ca/P: calcium/phosphorus ratio. Data are presented as median (25–75th interquartile range); a indicates median values outside reference range (internal laboratory reference values). Data in bold highlight statistical significance.

**Table 4:** Correlation (r) of all analytes with serum ferritin. Data in bold highlight statistical significance (red: negative correlation; blue: positive correlation).

Ferritin vs.	All Dogs (*n* = 92)	Lean/Normal Weight (CG, *n* = 55)	Overweight/Obese (OG, *n* = 37)
RBC	0.017	0.084	−0.159
Haemoglobin	0.081	0.027	0.074
Haematocrit	0.065	0.032	−0.169
MCV	0.094	−0.238	**0.448**
MCH	0.148	−0.017	**0.457**
MCHC	0.173	**0.273**	0.306
RDW	0.033	0.180	**−0.557**
WBC	−0.001	0.184	**−0.410**
Segmented	0.110	0.216	−0.339
Lymphocytes	−0.159	−0.086	−0.165
Monocytes	−0.018	0.090	−0.310
Eosinophiles	−0.102	0.096	**−0.492**
Basophiles	−0.117	−0.035	−0.310
Platelets	−0.210	−0.157	−0.251
MPV	−0.019	0.033	−0.285
PCT	−0.150	−0.007	−0.369
MPC	0.156	0.095	0.299
PCDW	0.038	**0.302**	**−0.473**
MPM	−0.005	0.044	−0.187
PMDW	0.033	0.082	−0.159
Total proteins	0.150	0.094	−0.044
Albumin	−0.036	−0.066	−0.226
Globulins	0.080	0.079	−0.146
CRP	−0.018	0.118	−0.155
Haptoglobin	−0.080	−0.037	−0.368
Fe	0.117	0.028	0.016
UIBC	−0.065	−0.083	−0.156
TIBC	0.082	−0.014	−0.177
Ferritin saturation	0.090	0.005	0.122
CK	0.102	0.146	−0.114
AST	**0.225**	0.187	0.187
ALT	0.161	0.141	0.126
FAL	0.136	−0.018	0.295
gGT	**−0.270**	**−0.303**	−0.008
Total bilirubin	0.132	0.034	0.331
BChE	0.192	0.081	0.084
PON-1	0.048	0.045	0.169
Cholesterol	0.024	−0.076	0.271
Triglycerides	0.154	0.130	−0.040
Amylase	0.164	0.204	0.081
Urea	0.099	0.020	0.352
Creatinine	0.152	0.147	0.166
Glucose	−0.202	−0.001	−0.207
Calcium	0.043	−0.051	−0.087
Phosphorus	−0.068	−0.139	0.004
Ca/P	0.080	0.055	−0.019

RBC: red blood cells; MCV: mean corpuscular volume; MCH: mean corpuscular haemoglobin; MCHC: mean cell haemoglobin concentration; WBC: white blood cell count; MPV: mean platelet volume; PCT: platelet crit; MPC: mean platelet component concentration; PMDW: platelet dry mass distribution width. CRP: C-reactive protein; Fe: iron; UIBC: unsaturated iron-binding capacity; TBIC: total ferric fixation capacity; CK: creatinine kinase; AST: aspartate aminotransferase; ALT: alanine aminotransferase; ALP: alkaline phosphatase; gGT: gamma glutamyl aminotransferase; BChE: butyrylcholinesterase; PON-1: paraoxonase-1; Ca/P: calcium/phosphorus ratio.

## Data Availability

The data presented in this study is available upon reasonable request to corresponding author.
